# *Squamarina* (lichenised fungi) species described from China belong to at least three unrelated genera

**DOI:** 10.3897/mycokeys.66.39057

**Published:** 2020-04-24

**Authors:** Yan-Yun Zhang, Xin-Yu Wang, Li-Juan Li, Christian Printzen, Einar Timdal, Dong-Ling Niu, An-Cheng Yin, Shi-Qiong Wang, Li-Song Wang

**Affiliations:** 1 CAS Key Laboratory for Plant Diversity and Biogeography of East Asia, Kunming Institute of Botany, Chinese Academy of Sciences, Kunming, Yunnan 650201, China; 2 University of Chinese Academy of Sciences, Beijing 100049, China; 3 Department of Botany and Molecular Evolution, Senckenberg Research Institute, 60325 Frankfurt am Main, Germany; 4 Natural History Museum, University of Oslo, P.O. Box 1172, Blindern, N-0318 Oslo, Norway; 5 Department of Life Science, Ningxia University, Yinchuan, Ningxia 750021, China

**Keywords:** Squamarinaceae, *
Petroplaca
*, *
Rhizoplaca
*, *
Lobothallia
*, *
Lecanora
*, type study

## Abstract

New collections of six *Squamarina* species from type localities in China were studied. The comparison of morphological characteristics and secondary metabolites with those of the type specimens and phylogenetic analyses suggest that *S. callichroa* and *S. pachyphylla* belong to *Rhizoplaca*, *S. semisterilis* belongs to *Lobothallia* and *S. chondroderma* should be retained in *Lecanora* temporarily. Only two species, *S. kansuensis* and *S. oleosa*, remain in *Squamarina*. The new combinations *Lobothallia semisterilis* (H. Magn.) Y. Y. Zhang, *Rhizoplaca callichroa* (Zahlbr.) Y. Y. Zhang and *R. pachyphylla* (H. Magn.) Y. Y. Zhang are proposed. Detailed descriptions to aid the identification of these species, distributions and phylogenetic trees, based on multiple collections, are presented. The generic concept of *Squamarina* is recircumscribed in this study.

## Introduction

The genus *Squamarina* Poelt was first erected by Poelt (1958) and is characterised by thick squamules, large apothecia and a “*Squamarina*-type” thallus, consisting of a well-separated and more or less equally high upper cortex, algal layer and medulla. Two sections, S. sect. Squamarina and S. sect. Petroplaca, were distinguished by Poelt (1958), based on the former having a larger thallus and larger apothecia, and the latter smaller thallus and apothecia. Hafellner (1984) accommodated the genus in a new family, Squamarinaceae, based on asci with an evenly amyloid tholus without any axial body. However, the circumscription of *Squamarina* or Squamarinaceae has been disputed for a long time and molecular studies for this genus are largely lacking (Hafellner 1984; Haugan and Timdal 1992; Hertel and Rambold 1988; Poelt 1958). Recent studies showed that the species of the sect. Squamarina have asci with an amyloid tube in the tholus, resembling those of Porpidiaceae and that the ascus structure of sect. Petroplaca resembles that of *Protoparmeliopsis muralis* (Schreb.) Rabenh. (Haugan and Timdal 1992; Hertel and Rambold 1988). Hence, the detailed circumscription of the genus *Squamarina* is urgently needed and it was also one of the aims of this study.

Nine species of *Squamarina* have so far been reported from China (Wei 1991), of which six were originally collected in China by Birger Bohlin and Heinrich Frh. von Handel-Mazzetti: *S. callichroa* (Zahlbr.) Poelt, *S. chondroderma* (Zahlbr.) Wei, *S. kansuensis* (H. Magn.) Poelt, *S. oleosa* (Zahlbr.) Poelt, *S. pachyphylla* (H. Magn.) Wei and *S. semisterilis* (H. Magn.) Wei. Although these species were published about 100 years ago (Magnusson 1940; Zahlbruckner 1930), no more collections have, however, been reported since then, except for *S. chondroderma* and molecular data are not available for any of them in GenBank. Therefore, studies on the identification, distribution and phylogeny of these species are necessary. We have undertaken several field trips along the collection routes of Birger Bohlin (1930–1932) and Handel-Mazzetti (1914–1915) in the past few years and collected fresh material of the six species from the type localities for the molecular study presented here.

## Methods

### Morphological and chemical studies

Type specimens were loaned from the Museum of Natural History Vienna (W) and the Swedish Museum of Natural History (S). The fresh material collected for this study is deposited in Kunming Institute of Botany, Chinese Academy of Sciences (KUN-L). Morphological features were studied under a dissecting microscope (Nikon SMZ745T). Apothecia and thalli were sectioned with an S-30 microtome with a KS-34 cryostat (Zeiss, Jena) and microscopic traits were observed and measured using a microscope (Leica 020-518.500). Secondary metabolites were analysed by spot reactions and thin-layer chromatography (TLC) in solvents A, B and C (Orange et al. 2001).

### DNA extraction, PCR and sequencing

Total DNA was extracted from dry or fresh specimens using the DNeasy Plant Mini Kit (Qiagen, Germany), according to the manufacturer’s instructions. Amplifications were performed in a 25 μl volume containing 12.5 μl 2 × MasterMix (TaqDNA Polymerase [0.1 units/μl], 0.4 mM MgCl_2_, 0.4 nM dNTPs) (Aidlab Biotechnologies Co. Ltd.), 0.5 μl of each primer, 10 μl ddH_2_O and 1 μl of DNA. The PCR settings and the primers of nrITS (ITS1-5.8S-ITS2), nrLSU, RPB1, RPB2 and mtSSU follow Zhao et al. (2015). All PCR reactions were sequenced by TsingKe Biological Technology (Kunming, China) using the amplification primers.

### Phylogenetic analyses

Sequences were assembled and edited using SeqMan 7.1 (DNAstar packages). An nrITS matrix of *Lobothallia* (Clauzade & Cl. Roux) Hafellner, an nrLSU matrix of *Squamarina* and a 5-locus (nrITS, nrLSU, RPB1, RPB2 and mtSSU) concatenated matrix of *Rhizoplaca* Zopf and related genera were generated using Geneious R8. Single-gene analyses were conducted, based on the Maximum Likelihood (ML) method to assess the conflict amongst individual genes and no significant incongruence was detected. Matrices were aligned with MAFFT, using the web service (http://mafft.cbrc.jp/alignment/server/index.html). Ambiguous positions were removed, using the web service of Guidance (http://guidance.tau.ac.il/ver2/). MrModeltest2.3 (Nylander 2004), based on Akaike Information Criterion (AIC), was used to estimate the best-fitting substitution model for each dataset for Maximum Likelihood (ML) and Bayesian Inference (BI). The selected model for nrITS-*Lobothallia* was HKY+I and, for the other matrices, GTR+I+G. Bayesian reconstructions of phylogenies were performed with MrBayes 3.1.2 (Huelsenbeck and Ronquist 2001), using four Markov chains running for 2 million generations for single locus matrices and 10 million generations for the concatenated dataset. Trees were sampled every 100 generations. ML analyses were performed with RaxmlHPC, using the General Time Reversible model of nucleotide substitution with the gamma model of rate heterogeneity (GTRGAMMA). Support values were inferred from the 70% majority-rule tree of all saved trees obtained from 1000 non-parametric bootstrap replicates. Trees were visualised in FigTree v1.4.0 (Rambaut 2012).

## Results and discussions

A total of 84 sequences of the nrITS, nrLSU, RPB1, RPB2 and mtSSU were newly generated for the species *Squamarina chondroderma*, *S. semisterilis*, *S. callichroa*, *S. pachyphylla*, *S. gypsacea* (Sm.) Poelt, *S. kansuensis* and *S. oleosa* in this study (Table 1). The BLAST results showed that these species belong to at least three unrelated genera, *Lobothallia*, *Squamarina* and *Rhizoplaca*, respectively. Given the large evolutionary divergence of these species, we reconstructed three separate phylogenies focusing on the three genera, based on nrITS, nrLSU and a 5-locus (nrITS, nrLSU, RPB1, RPB2 and mtSSU) concatenated matrix, respectively (Figs 2, 4, 6), to clarify the phylogenetic position of the six species. The results showed that *Squamarina semisterilis* is nested within the genus *Lobothallia*, which is closely related to the species *L. alphoplaca* (Wahlenb.) Hafellner, *L. melanaspis* (Ach.) Hafellner and *L. praeradiosa* (Nyl.) Hafellner, but differs in having a pruinose thallus and grows on soil. The *Aspicilia*-type ascus and bacilliform conidia clearly distinguish this species from the genus *Squamarina*. *Squamarina callichroa* and *S. pachyphylla* were nested within the *Rhizoplaca chrysoleuca* (Sm.) Zopf group. The exclusion of the two species from *Squamarina* is also supported by their *Lecanora*-type ascus and the orange or black apothecia. Therefore, the new combinations *Lobothallia semisterilis* (H. Magn.) Y. Y. Zhang, *Rhizoplaca callichroa* (Zahlbr.) Y. Y. Zhang and *R. pachyphylla* (H. Magn.) Y. Y. Zhang are proposed here.

**Table 1.** Specimens and DNA sequences for nrITS, nrLSU, RPB1, RPB2 and mtSSU used in this study, with the corresponding voucher information from GenBank indicated. Sequences, newly obtained in this study, are indicated in boldface.

Species	Locality*	Voucher specimens	Accession number*				
			nrITS	nrLSU	RPB1	RPB2	mtSSU
*Aspicilia cinerea*	Sweden	Nordin 6213 (UPS)	JF703115	–	–	–	–
*A. epiglypta*	Sweden	Nordin 6105 (UPS)	HQ259262	–	–	–	–
*Cladia aggregata*	Australia	HTL 19970f (F)	–	GQ500969	–	–	–
*C. deformis*	Australia	HTL 19994d (F)	–	GQ500967	–	–	–
*Cladonia digitata*	na	Ekman 3424 (BG)	–	AY756319	–	–	–
*C. stipitata*	na	AFTOL-ID 1657 (DUKE)	–	DQ973026	–	–	–
*C. sulcata*	Australia	HTL 19975i (F)	–	GQ500959	–	–	–
*Herteliana schuyleriana*	USA: North Carolina	1885671	–	MH887488	–	–	–
*H. taylorii*	na	Hertel 39599 (UPS)	–	AY756351	–	–	–
*Heterodea muelleri*	Australia	Elix 39643 (CANB)	–	GQ500962	–	–	–
*Lecanora achroa*	Thailand	Papong 6458 (F)	JN943714	na	JN987926	KT453937	JQ782663
*L. caesiorubella*	Australia	Lumbsch 19974k (F)	JN943728	JN939508	JN987920	na	na
*L. chondroderma* 1	China: Yunnan	16-54907 (KUN-L)	**MK778053**	**MK778013**	**MK766421**	**MK766441**	na
*L. chondroderma* 2	China: Xizang	16-52925 (KUN-L)	**MK778052**	**MK778012**	**MK766420**	**MK766440**	**MN192155**
*L. chondroderma* 3	China: Xizang	16-53527 (KUN-L)	**MK778056**	**MK778016**	**MK766423**	**MK766443**	**MN192156**
*L. chondroderma* 4	China: Yunnan	17-55591 (KUN-L)	**MK778057**	**MK778017**	**MK766424**	**MK766444**	na
*L. conizaeoides*	na	K. Molnar U0505/M (DUKE)	na	na	KJ766862	KJ766956	KJ766418
*L. contractula*	na	AFTOL-ID 877 (DUKE)	HQ650604	DQ986746	DQ986817	DQ992428	DQ986898
*L. dispersa*	USA: Illinois	Leavitt 12-002	KT453733	na	KT453888	KT453921	na
*L. farinacea*	Australia	Lumbsch 20003 (F)	JN943725	JN939513	JN987924	na	JQ782672
*L. flavopallida*	Australia	Lumbsch 19972d (F)	JN943723	JN939516	JN987925	KT453938	JQ782673
*L. formosa*	China: Xinjiang	ZX 20129045-2 (SDNU)	KT453771	KT453773	na	KT453978	KT453819
*L. hybocarpa*	na	Lumbsch s.n. (F)	EF105412	EF105421	EF105430	na	EF105417
*L. intricata*	na	U166 (GZU)	AF070022	DQ787345	na	na	DQ787346
*L. novomexicana*	USA	55026 (BRY-C)	HM577257	na	KU935390	KU935136	na
*L. polytropa*	na	AFTOL-ID 1798 (DUKE)	HQ650643	DQ986792	na	DQ992418	DQ986807
*L. saligna*	USA	Leavitt 5702 (BRY-C)	KU934539	na	KU935293	KU935036	na
*L. tropica*	Kenya	Lumbsch 19573a (F)	JN943718	JN939537	JN987936	na	na
*Lecidella carpathica*	China: Xinjiang	ZX 20140367-2 (SDNU)	KT453741	KT453784	KT453905	KT453944	KT453831
*L. stigmatea*	China: Xinjiang	ZX 20140838 (SDNU)	KT453766	KT453803	KT453918	KT453971	KT453849
*L. tumidula*	China: Xinjiang	ZX XL0009 (SDNU)	–	KT453810	–	–	–
*Lepraria bergensis*	na	Tonsberg 28875 (BG)	–	AY756324	–	–	–
*L. incana*	na	AFTOL-ID 1792 (DUKE)	–	DQ986795	–	–	–
*Lobothallia alphoplaca*	China	20117616 (SDNU)	JX499233	–	–	–	–
*L. alphoplaca*	China	20117646 (SDNU)	JX476025	–	–	–	–
*L. crassimarginata*	China	20122565 (SDNU)	JX476026	–	–	–	–
*L. crassimarginata*	China	20122583 (SDNU)	KC007439	–	–	–	–
*L. helanensis*	China	20122517 (SDNU)	JX476030	–	–	–	–
*L. helanensis*	China	20122791 (SDNU)	JX476031	–	–	–	–
*L. melanaspis*	Sweden	Nordin 6622 (UPS)	HQ259272	–	–	–	–
*L. melanaspis*	Norway	Owe-Larsson 8943a (UPS)	JF825524	–	–	–	–
*L. praeradiosa*	China	20126314 (SDNU)	JX499232	–	–	–	–
*L. praeradiosa*	China	20126613 (SDNU)	JX499234	–	–	–	–
*L. pruinosa*	China	20123278 (SDNU)	JX476028	–	–	–	–
*L. pruinosa*	China	20123630 (SDNU)	JX476027	–	–	–	–
*L. radiosa*	Sweden	Nordin 5889 (UPS)	JF703124	–	–	–	–
*L. recedens*	Sweden	Nordin 6035 (UPS)	HQ406807	–	–	–	–
*L. semisterilis*	China: Qinghai	18-59262 (KUN-L)	**MK778040**	**MK778009**	na	na	na
*L. semisterilis*	China: Qinghai	18-59322 (KUN-L)	**MK778039**	**MK778008**	**MK766413**	na	na
*L. semisterilis*	China: Qinghai	18-59345 (KUN-L)	**MK778042**	**MK778011**	**MK766415**	na	na
*L. semisterilis*	China: Gansu	18-59596 (KUN-L)	**MK778041**	**MK778010**	**MK766414**	na	na
*Metus conglomeratus*	Australia	HTL 19982b (F)	–	GQ500958	–	–	–
*Miriquidica complanata*	Poland: Karkonosze Mts	Szczepanska 935 (herb. Szczepanska)	KF562187	KF562179	KF601233	na	KR995349
*M. garovaglii*	Slovakia: Karpaty Mts	Szczepanska 538 (herb. Szczepanska)	KF562188	na	KF601234	na	na
*Mycoblastus affinis*	na	AFTOL-ID 1047 (DUKE)	na	KJ766601	na	KJ766958	na
*M. sanguinarius*	na	AFTOL-ID 196 (DUKE)	DQ782842	DQ912333	na	DQ782867	DQ912276
*Paralecia pratorum*	Italy	M-0045925 (M)	–	KP224503	–	–	–
*Pilophorus cereolus*	na	na	–	AY340559	–	–	–
*P. strumaticus*	na	na	–	AY340560	–	–	–
*Protoparmeliopsis achariana*	na	U525	na	DQ787341	DQ973051	DQ973088	DQ972976
*P. garovaglii*	USA	Leavitt 106 (BRY-C)	KU934546	na	KU935300	KU935043	na
*P. muralis*	na	K. Molnar U0501/AO (EGR)	na	KJ766634	KJ766830	KJ766943	KJ766466
*P. peltata*	Iran	MS014622	KT453723	na	KT453892	KT453927	na
*P. zareii*	Iran	SK 480	KP059049	na	na	na	KP059055
*Ramboldia gowardiana*	na	Bjork 9447 (UBC)	na	KJ766649	KJ766889	na	KJ766483
*R. sanguinolenta*	Australia: Queensland	Elix 28835 (F)	EU075548	EU075523	KT453920	na	EU075534
*Rhizoplaca callichroa* 1	China: Sichuan	14-43348 (KUN-L)	**MK778045**	na	na	na	na
*R. callichroa* 2	China: Sichuan	14-43357 (KUN-L)	**MK778046**	na	na	na	na
*R. callichroa* 3	China: Sichuan	14-43359 (KUN-L)	**MK778043**	na	na	na	na
*R. callichroa* 4	China: Yunnan	14-43308 (KUN-L)	**MK778044**	na	na	na	na
*R. chrysoleuca* 1	USA	55000 (BRY-C)	HM577233	KT453812	KU935335	KU935084	KT453856
*R. chrysoleuca* 2	Iran	MS014636	KT453731	na	KT453898	KT453934	na
*R. huashanensis*	China	Wei 18357 (HAMS)	AY530885	AY648104	na	na	na
*R. marginalis* 1	USA: California	Leavitt 739 (BRY-C)	KT453732	na	KT453901	KT453936	na
*R. marginalis* 2	USA	0020826b (BRY-L)	KU934655	na	KU935370	KU935123	na
*R. melanophthalma*	Iran	MS014628 (H)	JX948271	na	JX948317	JX948355	na
*R. pachyphylla* 1	China: Gansu	18-59466 (KUN-L)	**MK778048**	na	**MK766417**	**MK766436**	**MN192152**
*R. pachyphylla* 2	China: Gansu	18-59446 (KUN-L)	**MK778047**	na	**MK766416**	**MK766435**	**MN192151**
*R. pachyphylla* 3	China: Gansu	18-59482 (KUN-L)	**MK778049**	na	**MK766416**	**MK766437**	**MN192153**
*R. pachyphylla* 4	China: Gansu	18-59561 (KUN-L)	**MK778050**	na	**MK766419**	**MK766438**	**MN192154**
*R. polymorpha*	USA	55095 (BRY-C)	HM577326	na	KU935411	KU935159	na
*R. porterii*	USA	55149 (BRY-C)	HM577380	na	JX948341	JX948380	na
*R. shushanii*	USA	55065 (BRY-C)	HM577286	na	JX948334	JX948372	na
*R. subdiscrepans*	Russia	Vondrak 9408 (PRA)	KU934898	na	KU935435	KU935187	na
*Squamarina cartilaginea*	na	AFTOL-ID 1281	–	DQ986763	–	–	–
*S. gypsacea*	Greece	O-L-196249 (O)	na	**MK778021**	na	na	na
*S. gypsacea*	Greece	O-L-196255 (O)	na	**MK778020**	na	na	na
*S. gypsacea*	Greece	O-L-59266 (O)	na	**MK778019**	na	na	na
*S. gypsacea*	Spain	O-L-16444 (O)	na	**MK778022**	na	na	na
*S. kansuensis*	China: Xizang	16-54052 (KUN-L)	**MK778059**	**MK778023**	**MK766425**	**MK766446**	na
*S. kansuensis*	China: Ningxia	14-09-1429 (NXAC)	**MK778060**	**MK778024**	**MK766426**	**MK766447**	na
*S. kansuensis*	China: Xinjiang	20139103 (XJU)	**MK778061**	**MK778025**	**MK766427**	**MK766448**	na
*S. kansuensis*	China: Qinghai	18-59260 (KUN-L)	**MK778062**	**MK778026**	**MK766428**	**MK766449**	na
*S. kansuensis*	China: Gansu	18-59601 (KUN-L)	na	**MK778031**	na	na	na
*S. lentigera*	na	Haugan & Timdal 4801 (O)	–	AY756363	–	–	–
*S. oleosa*	China: Yunnan	19-66398 (KUN-L)	**MN904892**	**MN904896**	na	**MN923191**	**MN915135**
*S. oleosa*	China: Yunnan	19-66399 (KUN-L)	**MN904893**	**MN904897**	**MN923189**	**MN923192**	**MN911318**
*S. oleosa*	China: Yunnan	19-66401 (KUN-L)	**MN904894**	**MN904898**	**MN923190**	**MN923193**	**MN915136**
*Stereocaulon alpinum*	Austria	AT1194 (HBG)	–	JN941201	–	–	–
*S. sasakii*	Japan	AT1187 (TUR)	–	JN941206	–	–	–
*S. tomentosum*	Finland	AT1084 (TUR)	–	JN941203	–	–	–

*na = not available; *– = not used in this study

*Lecanora chondroderma* (= *Squamarina chondroderma*) is sister to the genus *Rhizoplaca*, but differs in growing on moss and meadow and the presence of numerous rhizinose strands that are never present in its related genera. It is also distinct from the genus *Squamarina* by the *Lecanora*-type ascus and the strongly gelatinised lower cortex. This species could belong to a genus separate from *Lecanora* s. str. and closely related to the genera *Rhizoplaca* and *Protoparmeliopsis*, but as only one species from this group was included here, further exploration is needed in the future and we prefer to retain this species in *Lecanora* here. The remaining two species, *Squamarina kansuensis* and *S. oleosa*, proved to belong in *Squamarina*. *Squamarina kansuensis* is sister to *S. lentigera*, but differs in the larger thallus and the presence of psoromic and 2’-O-demethylpsoromic acids. *Squamarina oleosa* is a basal clade of the genus, which is close to the species *S. cartilaginea* (With.) P. James and *S. gypsacea*.

We revised the previously reported ascus structure for the two sections of *Squamarina* (Haugan and Timdal 1992; Hertel and Rambold 1988) and verified that the species in sect. Squamarina display a Porpidia -type ascus and the species in sect. Petroplaca form a *Lecanora*-type ascus. Our phylogenetic analyses, containing the type species of the two sections, *S. callichroa* and *S. gypsacea*, were in accordance with the ascus type: the sect. Squamarina is close to the genus *Stereocaulon* (Schreb.) Schrad., which also has a *Porpidia*-type ascus (Högnabba 2006); section Petroplaca is nested within the genus *Rhizoplaca* having a *Lecanora*-type ascus. Therefore, we suggest excluding the section Petroplaca from the genus *Squamarina* and recircumscribe this genus as follows: thallus saxicolous or terricolous, squamulose, placodioid or subfoliose, squamules or lobes dispersed, continuous to irregularly overlapping, very thick, usually with a white, thickened and slightly upturned marginal rim; upper surface white, yellowish-green, grey green to olive green, smooth to strongly cracked and wrinkled; lower surface white, pale brown to blackish-brown, well defined but without cortex; thallus section with well-differentiated upper cortex, algae layer and medulla; upper cortex with pale brown granules, turning colourless in potassium hydroxide (KOH); algal layer continuous; medulla very thick, filled with grey calcium oxalate crystals that become needle shaped after treatment with 25% sulphuric acid (H_2_SO_4_); apothecia lecanorine type, algal layer usually absent from the margin and only present under hypothecium, rarely biatorine type because of the strong convex disc; disc light yellow, yellow, pale brown to reddish-brown, pruinose or not; ascus narrowly clavate, *Porpidia*-type, 8-spored; ascospores colourless, ellipsoid to subfusiform, non-septate; pycnidia yellowish-brown, conidia filiform, curved; usnic acid always present and psoromic acid also present in most species.

### Taxonomy

#### Lobothallia semisterilis

Taxon classificationFungiLecanoralesStereocaulaceae

(H. Magn.) Y. Y. Zhang comb. nov.

8F5904F2-BA30-5908-8010-35B472B099C7

832199

Fig. 1A–E

Lecanora semisterilis H. Magn., Lichens from Central Asia 1: 123–124 (1940) (Basionym). ≡ Squamarina semisterilis (H. Magn.) J.C. Wei, Enumeration of Lichens in China: 232 (1991). Type: China, Gansu Province, 2450–2600 m elev., on soil, 1931, Birger Bohlin 38L (S–Holotype!).

##### Description.

Thallus to 5 cm across, areolate centrally, with irregularly elongate lobes at the margin, closely to loosely attached to soil; areoles angular, plane to slightly convex, continuous to crowed, ca. 1 mm across; marginal lobes ca. 1 mm wide and 2–3 mm long; upper surface white to grey, pruinose, the pruina on the marginal lobes becoming granular; lower surface white, attached to soil directly with medullary hyphae. Upper cortex colourless with pale brown upper part, 22–55 μm high; epinecral layer colourless, 10–20 μm high; algal layer ca. 95 μm high, not continuous, the interval between different groups of algae 16–32 μm wide; medulla filled with grey granules, lower cortex lacking.

Apothecia rounded, sessile, constricted at the base, up to 2 mm in diam.; disc plane to slightly convex, blackish-brown, non-pruinose; thalline margin entire, concolorous with thallus; hymenium colourless, ca. 60 μm high; subhymenium and hypothecium colourless, I + blue; epihymenium consisting of brown granules, ca. 15 μm high; paraphyses simple, slightly thickened at the apex, ca. 3 μm in diam.; asci *Aspicilia*-type, 8-spored; ascospores colourless, ellipsoid, 9–13 × 5–9 μm.

Pycnidia prominent, sometimes protruding from the thallus-like apothecia, with blackish-brown ostioles, numerous, 0.1–0.4 mm across; conidia bacilliform, 5.5–6.5 × ca. 1 μm.

**Figure 1.**
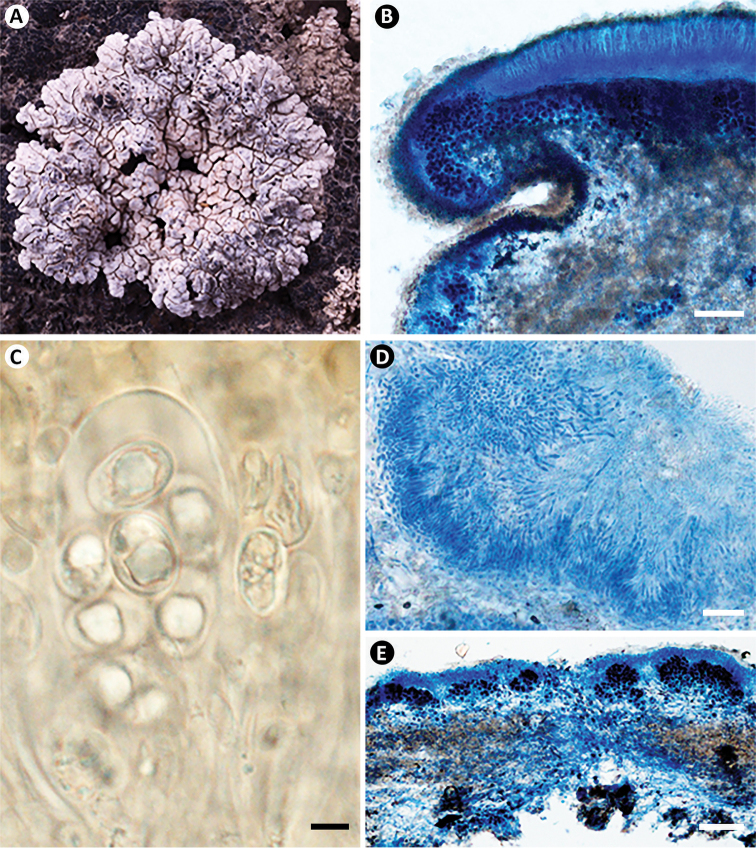
*Lobothallia semisterilis* (KUN-L 18-59656). **A** Habit **B** apothecial anatomy (LCB) **C** ascus and spores (Lugol’s) **D** section of pycnidia (LCB) **E** section of thallus (LCB). Scale bars: 100 μm (**B, E**); 5 μm (**C**); 20 μm (**D**).

##### Chemistry.

Upper cortex K + red, C-, P-, medulla K + red, C-, P + yellow; norstictic acid.

##### Ecology and distribution.

Growing on soil in very dry habitats at elevations of 1760–3151 m. This species was previously only known from Gansu Prov. and is reported here as new to Qinghai Prov., China.

##### Notes.

The holotype consists of numerous fragments on soil, without apothecia but numerous pycnidia. This species was originally described as a *Lecanora* by Magnusson (1940) and transferred to *Squamarina* by Wei (1991). We initially treated our materials as “*S. semisterilis*” since their morphology was identical with the holotype, which is characterised by the pruinose and lobate thallus containing norstictic acid, terricolous habit, pycnidia resembling apothecia and bacilliform conidia. We transfer this species to the genus *Lobothallia*, based on the phylogenetic reconstruction. Its position within this genus is supported by the lobate and slightly convex thallus, the *Aspicilia*-type ascus, the bacilliform conidia and the absence of usnic acid.

**Figure 2.**
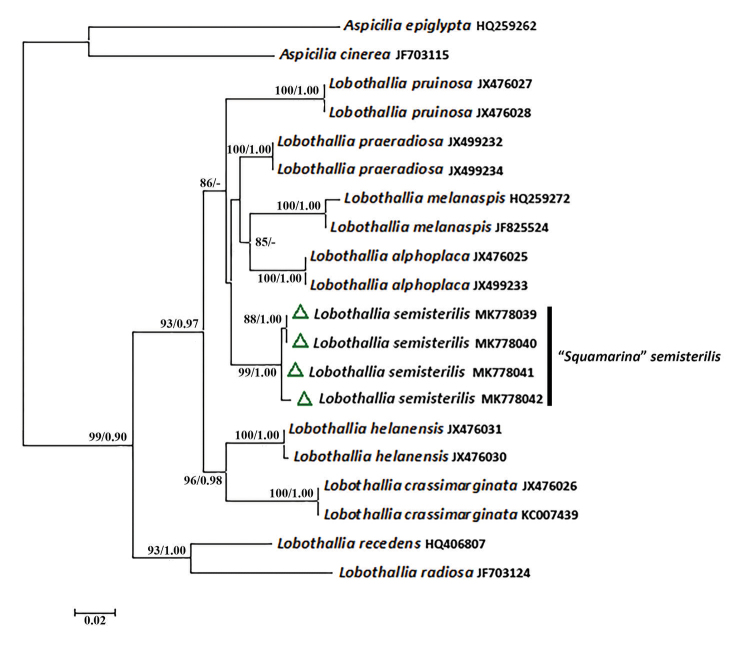
Maximum Likelihood phylogeny of the genus *Lobothallia*, based on nrITS. ML bootstrap value ≥ 70% and posterior probabilities ≥ 0.95 from the Bayesian analysis are given adjacent to nodes.

The genus *Lobothallia* is a small genus mainly growing on rocks, containing twelve species (Kou et al. 2013; Lücking et al. 2017). We added eight of these species as intergroups to assess the phylogenetic position of *Lobothallia semisterilis* in the genus. The results show that *Lobothallia semisterilis* is close to *L. alphoplaca*, *L. melanaspis* and *L. praeradiosa* in the phylogeny (Fig. 2). However, *L. alphoplaca* differs in the epruinose thallus and the presence of constictic and stictic acids, *L. melanaspis* differs in the saxicolous habit and the distinctly rosette-forming thallus. *L. praeradiosa* can be distinguished by the epruinose and green grey to orange brown thallus (Galloway and Ledingham 2012; Kou et al. 2013). *Lobothallia pruinosa* Kou & Q. Ren is similar to *L. semisterilis* in having a pruinose upper surface, but differs in the saxicolous habit and the presence of constictic acid (Kou et al. 2013).

#### Rhizoplaca callichroa

Taxon classificationFungiLecanoralesStereocaulaceae

(Zahlbr.) Y. Y. Zhang comb. nov.

35399DEA-EE01-5172-BDAC-599017942ABF

832200

Fig. 3A–D

Lecanora callichroa Zahlbr., in Handel-Mazzetti, Symb. Sinic. 3: 172–173 (1930) (Basionym) ≡ Squamarina callichroa (Zahlbr.) Poelt, Mitt. Bot. Staatssamml., München 1–20: 527 (1958). Type: China, Yunnan Province, 2100 m elev., on rock, 1914, Heinrich Frh. von Handel-Mazzetti 35 (W–Isotype!)

##### Description.

Thallus saxicolous, to 4 cm across, squamulose to placodioid; squamules pruinose on the edges, more or less umbilicate when young; central squamules scattered to continuous, closely attached to the substrate, 1–2 mm across; marginal squamules larger than those in the centre, 2–4 mm across, with 1–2 mm free margin; upper surface yellowish-brown, smooth, plane to slightly convex; lower surface pale to pale brown, without rhizinose strands. Upper cortex filled with yellowish-brown granules dissolving in KOH, ca. 32 μm high; epinecral layer also filled with yellowish-brown granules, ca. 15 μm; algal layer continuous, 64–80 μm high; medulla thick, filled with grey to pale brown granules; lower cortex of free margin poorly developed, non-gelatinised, ca. 30 μm.

Apothecia lecanorine, laminal, dispersed, sessile, becoming slightly constricted at the base, round to irregular, 0.5–1.5 mm; disc orange, covered with pale pruina, plane to slightly convex; thalline margin entire and thick when young, becoming thin and occasionally flexuose with age; hymenium with scattered orange granules, I+ blue, ca. 80 μm high; thalline margin with evenly thick cortex, ca. 26 μm thick; epihymenium yellowish-brown, ca. 10 μm high; subhymenium and hypothecium colourless; ascus *Lecanora*-type, 8-spored; paraphyses slightly branched, without anastomoses; ascospores subfusiform to ellipsoid, 9.5–13.5 × 6–9 μm. Pycnidia immersed in the thallus, with pale brown ostioles; conidia filiform, straight to slightly curved, 19–26 × ca. 0.7 μm.

**Figure 3.**
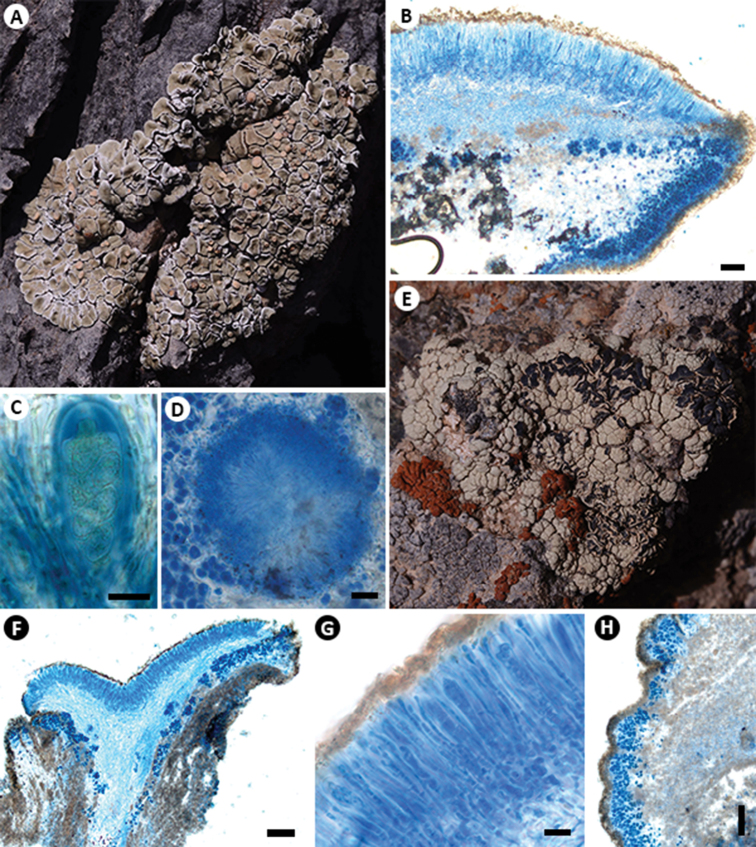
*Rhizoplaca callichroa* (**A–D**KUN-L 19-62900): **A** habit **B** apothecial anatomy (LCB) **C** ascus and spores (Lugol’s) **D** section of pycnidia (LCB). *R. pachyphylla* (**E–H**KUN-L 18-59446): **E** habit **F** section of apothecia **G** ascus and spores (LCB) **H** section of thallus (LCB). Scale bars: 100 μm (**B, F, H**); 10 μm (**C, G**); 20 μm (**D**).

##### Chemistry.

Upper cortex K-, C-, P-, medulla K + yellow, C-, P-; usnic and placodiolic acids.

##### Ecology and distribution.

Growing on rock in arid environments at elevations of 984–2100 m. Previously only known from Yunnan Prov., here reported as new to Sichuan Prov., China.

##### Notes.

**Figure 4.**
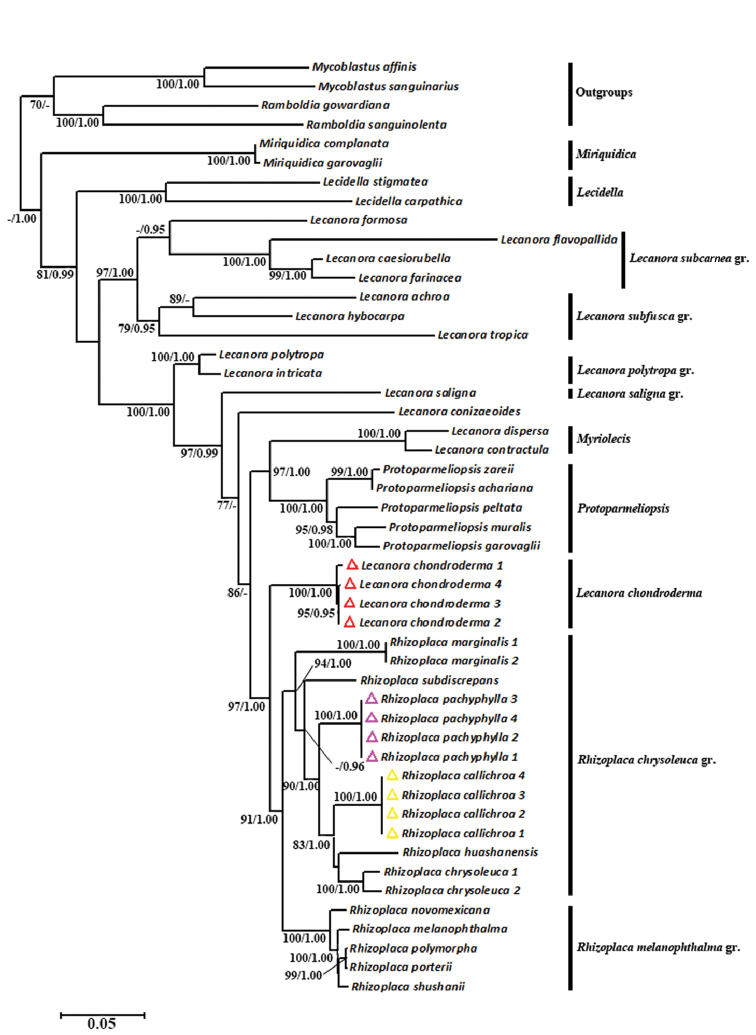
Maximum Likelihood phylogeny of the genus *Rhizoplaca* and related genera of Lecanoraceae, based on combined nrITS, nrLSU, RPB1, RPB2 and mtSSU. ML bootstrap value ≥ 70% and posterior probabilities ≥ 0.95 from the Bayesian analysis are given adjacent to nodes.

The isotype grows on quartzitic rock ca. 2 cm across, containing several intact apothecia. The spore size of “*Squamarina callichroa*”, given in the protologue, is 15–20 × 8–9 μm (Zahlbruckner 1930); however, Poelt (1958) measured the spore size of the type material as 11–12 × 8–9 μm. Our measurements of the freshly collected materials, 9.5–13.5 × 6–9 μm, are in accordance with Poelt’s results and the other characteristics, elevation and locality of our collections are more or less identical with the isotype. We did not find any specimens around the type locality having those long ascospores as in the description of the protologue. Therefore, we treat our specimens as “*Squamarina callichroa*” .This species was originally described as a *Lecanora* by Zahlbruckner (1930) and transferred to *Squamarina* as the type species of the section Petroplaca by Poelt (1958). We transfer this species to the genus *Rhizoplaca*, primarily based on its nested position within the *R. chrysoleuca* group in the phylogeny (Fig. 4) and also based on the orange apothecia, the *Lecanora*-type ascus and the presence of usnic and placodiolic acids. The genus *Rhizoplaca* is a small genus containing eleven species (Lücking et al. 2017). We added nine of these species as intergroups to assess the phylogenetic position of *R. callichroa* in the genus. The results show that *R. callichroa* is sister to *R. chrysoleuca* and *R. huashanensis* J.C. Wei, which differ by the umbilicate thallus, narrower ascospores, (7)8.5–12 × 3.5–6 μm and the monophyllus thallus and black apothecia, respectively (Nash et al. 2002; Wei 1984). *Rhizoplaca subdiscrepans* (Nyl.) R. Sant. is similar to *R. callichroa* in the squamulose thallus and orange apothecia, but differs in the very convex and smaller (0.3–1 mm) squamules and the narrower ascospores 7–12 × 3.5–5 μm.

##### Specimens examined (KUN-L).

China: Sichuan Province: Huili Co., beside Jiaopingdu bridge, near to the Jinsha river, 1550 m elev., 26°18'N, 102°22'E, on rock, 2014, Li-Song Wang et al. 14-43348, 14-43357, 14-43359; Yunnan Province: Luquan Co., beside Jiaopingdu bridge, 984 m elev., 26°18'N, 102°22'E, on rock, 2014, Li-Song Wang et al. 14-43308.

#### Rhizoplaca pachyphylla

Taxon classificationFungiLecanoralesStereocaulaceae

(H. Magn.) Y. Y. Zhang comb. nov.

AE28E860-0178-5E24-B986-BE3D0AC7F726

832201

Fig. 3E–H

Lecanora pachyphylla H. Magn., Lichens from Central Asia 1: 120–121 (1940) (Basionym) ≡ Squamarina pachyphylla (H. Magn.) J.C. Wei, Enumeration of Lichens in China: 232 (1991).Type: China, Gansu Province, 3800–3850 m elev., on rock, 1932, Birger Bohlin (S–Holotype!).

##### Description.

Thallus saxicolous, areolate without lobate margin, to 4 cm across, to 5 mm thick; areoles continuous, plane to slightly convex, 1–2 mm across; upper surface yellow, densely shallow rimose; lower side with thick, grey to white hypothallus. Upper cortex uneven, filled with yellowish-brown granules dissolving in KOH, 32–48 μm thick, algal layer continuous, variable in height, 80–128 μm; medulla very thick, filled with grey to pale brown granules; lower cortex lacking.

Apothecia common, usually densely grouped, irregular in shape, up to 5 mm in diam.; disc black, pruinose at the centre, plane when young, strongly concave with age; thalline margin thin and crenate, strongly bending towards inside with age; hymenium colourless, I+ blue, ca. 50 μm high; epihymenium containing yellowish-brown granules, ca. 9.5 μm high; subhymenium and hypothecium colourless; paraphyses evenly septate, simple, 2–3 μm in diam., apex more or less swollen and bluish-green, ca. 4.5 μm in diam.; ascus *Lecanora*-type, 8-spored; ascospores regular in shape, ellipsoid, colourless, 5.8–8 × 3–4.5 μm.

##### Chemistry.

Upper cortex K-, C-, P-, medulla K-, C-, P-; usnic acid and traces of unknown substances.

##### Ecology and distribution.

Growing on rock at elevations of 3291–3909 m. Only known from Gansu Prov., China.

##### Notes.

The holotype grows on rock with *Lecidea tessellata* Flörke, *Lecanora asiatica* H. Magn. and *Xanthoria elegans* (Link) Th. Fr. and contains numerous apothecia.

This species was originally described as a *Lecanora* by Magnusson (1940) and transferred to *Squamarina* by Wei (1991). It is characterised by the yellowish, areolate and very thick thallus, the black lecanorine apothecia and the very small ascospores. We transfer this species to *Rhizoplaca*, primarily based on the phylogenetic results (Fig. 4) and also based on the yellow thallus, the large, concave apothecia with margins bending towards the inside and the *Lecanora*-type ascus. *Rhizoplaca pachyphylla* is phylogenetically closely related to *R. callichroa*, *R. chrysoleuca* and *R. huashanensis*, but differs in the very thick and areolate thallus without lobate margin and the very small ascospores, 5.8–8 × 3–4.5 μm. *Rhizoplaca subdiscrepans* is similar to *R. pachyphylla* in the squamulose thallus, but differs in the orange apothecia, longer ascospores, 7–12 × 3.5–4.5 μm, and the presence of pseudoplacodiolic or placodiolic acids. *Rhizoplaca melanophthalma* (DC.) Leuckert is also similar to the species in having black apothecia, but differs in the umbilicate thallus and the larger ascospores, 6.5–12 × 4–7 μm.

##### Specimens examined (KUN-L).

China: Gansu Province: Shubei Co., Mengke Glacier, 3942 m elev., 39°12'N, 95°23'E, on rock, 2018, Li-Song Wang et al. 18-59446, 18-59466, 3785 m elev., on rock, 2018, Li-Song Wang et al. 18-59482; Yumen Ci., Yuerhong Vi., 3291 m elev., 39°50'N, 96°45'E, on rock, 2018, Li-Song Wang et al. 18-59560, 18-59561.

#### Lecanora chondroderma

Taxon classificationFungiLecanoralesStereocaulaceae

Zahlbr., in Handel-Mazzetti, Symb. Sinic. 3: 174 (1930).

B450ABEE-FB9D-5232-BB73-18974E9AC3E3

Fig. 5A, B

≡ Squamarina chondroderma (Zahlbr.) J.C. Wei, Enumeration of Lichens in China: 231 (1991).Type: China, Sichuan Province, 3600–3900 m elev., 1914, Heinrich Frh. von Handel-Mazzetti 497 (W–holotype!)

##### Description.

Thallus to 6 cm across, squamulose or lobate, growing on moss over rock or on the meadow; squamules 0.5–2 mm across, convex, continuous to slightly overlapped; marginal lobes branched, convex, 0.5–2 mm wide, 2–4 mm long; the apex of squamules and lobes rounded, bent downwards; upper surface smooth, pale green to straw, covered by white pruina; lower surface pale to dark brown in the centre and white to pale brown at the margin; rhizinose strands blackish-brown. Upper cortex very thin, ca. 16 μm, filled with yellowish-brown granules dissolving in KOH; algal layer continuous, 48–60 μm thick, medulla filled with grey to pale brown granules, 129–161 μm high, medullary hyphae very loose, more or less hollow in centre; lower cortex well separated from medulla, evenly thick with strongly gelatinised and anticlinally arranged hyphae, ca. 80 μm thick, colourless, hyphae at lower part brown. Apothecia lecanorine, sessile, with constricted base, rounded, scattered or in small groups, up to 3 mm in diam.; disc pruinose, reddish to dark brown, slightly concave when young, slightly convex with age; thalline margin concolorous with thallus, entire to flexuose, forming a well-delimited cortex consisting of strongly gelatinised and anticlinally arranged hyphae; hymenium colourless, 58–80 μm; epihymenium filled with yellowish-brown granules, 10–15 μm; paraphyses simple, evenly septate; ascus *Lecanora*-type, 8-spored; ascospores colourless, ellipsoid to slightly ovoid, 7–13 × 6.5–9 μm.

**Figure 5.**
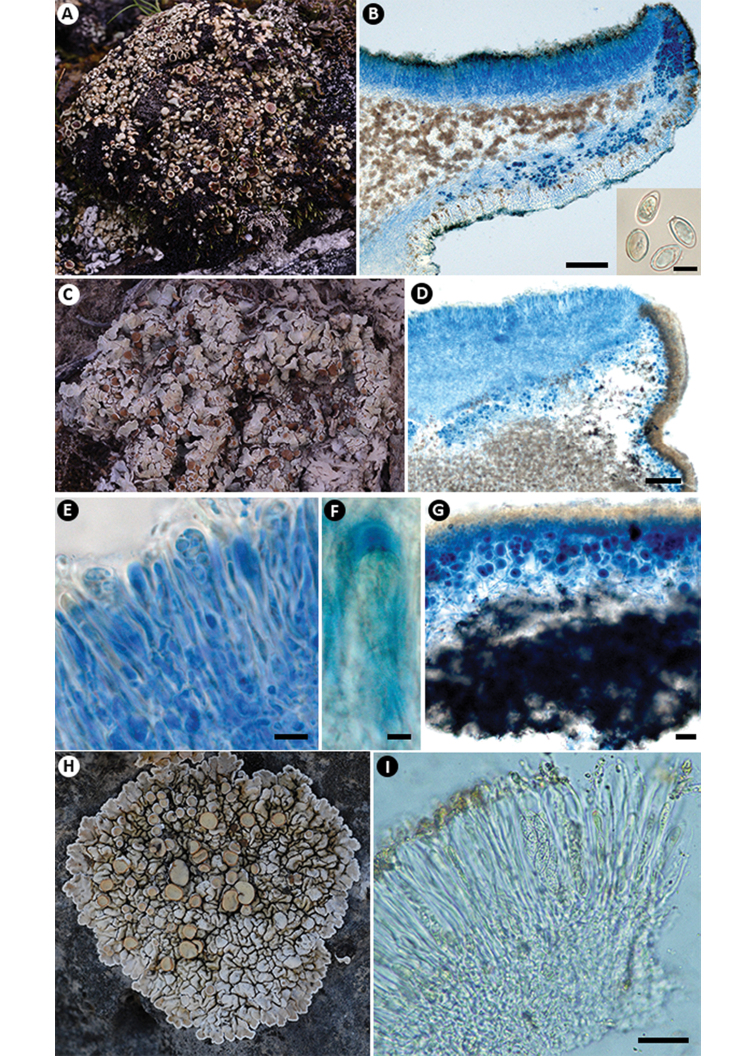
*Lecanora chondroderma* (**A, B**KUN-L 18-60317): **A** habit **B** apothecial anatomy (LCB) and ascospores (water). *Squamarina kansuensis* (**C–G**KUN-L 18-59601): **C** habit **D** apothecial anatomy (LCB) **E** ascus and ascospores (LCB) **F** apical structure of ascus (Lugol’s) **G** section of thallus (LCB). *S. oleosa* (**H, I**KUN-L 09−30043): **H** habit **I** ascus and ascospores (water). Scale bars: 100 μm (**B**-apothecia, **D**); 5 μm (**B**-ascospores, **F**); 10 μm (**E**); 20 μm (**G**); 25 μm (**I**).

##### Chemistry.

Upper cortex K-, C-, P + yellow, medulla K+ yellow, C-, P-; usnic acid and zeorin present in each sample, placodiolic and isousnic acids also present in most samples.

##### Ecology and distribution.

Growing on moss over rock or in meadow at 3600–4968 m elevation in the alpine zone. Worldwide distribution: China, India and Nepal. China: Sichuan Prov., reported here as new to Yunnan and Xizang provinces.

##### Notes.

The holotype of *Lecanora chondroderma* consists of several fragments, containing numerous apothecia.

*Lecanora chondroderma* was originally described by Zahlbruckner (1930) and transferred to *Squamarina* by Wei (1991). We transfer this species back to *Lecanora* temporarily because of its *Lecanora*-type ascus and phylogenetic position being closely related to the genera *Rhizoplaca* and *Protoparmeliopsis* (Fig. 4). Although *Lecanora chondroderma* is highly supported as a basal clade of the genus *Rhizoplaca* in our topology, it differs in dwelling on moss and meadow and having numerous rhizinose strands. Given that there are still many taxa of *Lecanora* which have not been included in our analyses and the phylogenetic relationships between *Rhizoplaca* and its related genera have still not been thoroughly resolved, we prefer to retain this species in *Lecanora* temporarily, rather than treat it as *Rhizoplaca*. *Lecanora chondroderma* is only known from the Himalayan region at elevations between 3600–4968 m. The morphology of the species varies amongst localities, with samples growing on moss over rock in Yunnan and Sichuan provinces, having broad (1–2 mm) and pale green lobes and samples from meadows at higher altitudes in Xizang Prov. developing narrower (0.5–1 mm) and more branched lobes with a yellowish appearance. These populations, however, share a pruinose thallus, convex lobes with rounded and downwards bent apices, a loose medulla, a well-delimited cortex of the thalline margin and lower cortex and the presence of usnic acid and zeorin.

*Lecanora geophila* (Th. Fr.) Poelt is similar to *L. chondroderma* in morphology, chemistry and habitat, whereas the former forms a yellowish crustose, squamulose to placodioid thallus with loboid projections or phyllidia or terete lobes and epruinose, pale, flat to convex apothecia, including usnic acid, zeorin and methylplacodiolic acid (Brodo 1981; Obermayer and Kantvilas 2003); the latter presents a totally pruinose, squamulose to lobate thallus that never forms phyllidia and terete lobes, pruinose, reddish-brown to black apothecia, numerous rhizinose strands and absence of methylplacodiolic acid.

##### Specimens examined (all in KUN-L unless otherwise noted).

China: Sichuan Province: 4650 m elev., 1915, Heinr. Frh. & Handel-Mazzetti 1353 (W). Yunnan Province: Shangri-La Co., Mt. Hong Shan, 4470 m elev., 28°07'N, 99°54'E, on soil, 2018, Li-Song Wang et al. 18-60317; Luquan Co., Mt. Jiaozi Snow, 4000 m elev., 26°05'N, 102°51'E, on moss over rock, 2016, Li-Song Wang et al. 16-54907; Lijiang Co., Mt. Laojunshan, 4036 m elev., 26°37'N, 99°44'E, on rock, 2017, Li-Song Wang et al. 17-55591. Xizang Province: Linzhou Co., Mt. Qiala, 4830 m elev., 30°06'N, 91°16'E, on the meadow, 2016, Li-Song Wang et al. 16-53527; Zuogong Co., on the way from Rumei to Zuogong, 4968 m elev., 29°43'N, 98°01'E, on the meadow, 2016, Li-Song Wang et al. 16-52925, 16-53079, on the meadow, 2016, Li-Song Wang et al. 16-52931.

#### Squamarina kansuensis

Taxon classificationFungiLecanoralesStereocaulaceae

(H. Magn.) Poelt

44FA90A8-0760-53C1-B3F0-EDC541F92ABA

Fig. 5C–G

Lecanora kansuensis H. Magn., Lichens from Central Asia 1: 116–117 (1940). Type: China, Gansu Province, 1500–1700 m elev., on soil, 1930, Birger Bohlin 20_2_ (S–Holotype!) (Basionym)

##### Description.

Thallus terricolous, loosely to tightly adnate on soil, irregular to radiate in outline and with elongate marginal lobes, up to 10 cm in diam.; lobes 2–4(5) mm long, 1–2(3) mm wide, 0.2–0.4 mm thick, with white, thickened and slightly upturned edges, more or less overlapping; upper surface greenish to straw, pruinose and strongly cracked at least in the centre of the thallus; lower surface well delimited, milk-white to pale, without rhizines, margins usually containing sparse white tomentum. Upper cortex filled with yellowish-brown granules, turning colourless in KOH, 26–32 μm thick; epinecral layer grey to brown, 5–15 μm thick; algal layer continuous, well delimited, ca. 50 μm high; medulla grey, filled with calcium oxalate crystals; lower cortex lacking.

Apothecia frequent, rounded, single or in small groups, usually less than 2 mm in diam. Disc pale brown to reddish-brown, slightly concave to flat when young, usually becoming strongly convex with age. Thalline margin distinctive when young and disappearing with age. Hymenium colourless, I + blue, ca. 65 μm high; epihymenium yellowish-brown turning colourless in KOH, ca. 12.5 μm high; thalline margin with evenly thick cortex filled with grey granules; paraphyses septate, ca. 2.5 μm in diam.; hypothecium colourless, 75–87 μm high; algal layer below hypothecium continuous, 62–87 μm high; ascus *Porpidia*-type, 8-spored; ascospores colourless, ellipsoid to slightly fusiform, variable in size and shape even within one ascus, 7.5–15 × 5–7.5 μm.

##### Chemistry.

Upper cortex K-, C-, P-, medulla K-, C-, P+ yellow; isousnic, usnic, psoromic and 2’-O-demethylpsoromic acids.

##### Ecology and distribution.

Growing on soil at 1310–4730 m of elevation. Previously only known from Gansu Prov. and reported here as new to Neimenggu, Ningxia, Sichuan, Xizang, Xinjiang and Yunnan provinces, China.

##### Notes.

The holotype consists of several small fragments on soil, bearing a single small apothecium. This species was originally described as a *Lecanora* by Magnusson (1940) and transferred to *Squamarina* by Poelt (1958). It is characterised by the pruinose, greenish- to straw-coloured thallus, lobes with white, thickened and slightly upturned edges, exposing a milk-white to pale lower surface, without rhizines and the presence of psoromic and 2’-O-demethypsoromic acids. This species is very common in the deserts and alpine zones of China. In desert regions, the thallus is usually irregular in outline with wider lobes and becomes rosette-like with narrower lobes when growing in the alpine zone.

**Figure 6.**
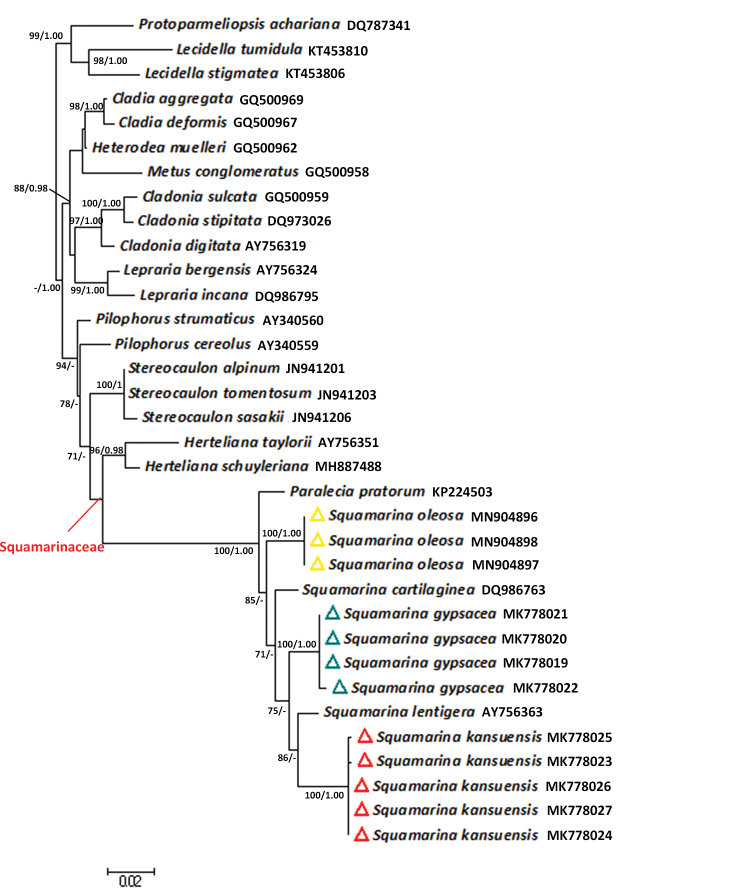
Maximum Likelihood phylogeny of the genus *Squamarina* and related genera, based on nrLSU. ML bootstrap value ≥ 70% and posterior probabilities ≥ 0.95 from the Bayesian analysis are given adjacent to nodes.

The genus *Squamarina* (= S. sect. Squamarina) includes eleven species (Poelt 1958) and there are three species with sequences in GenBank. We integrated the data from GenBank with the newly-produced data here to reconstruct the phylogeny of the genus *Squamarina* to assess the phylogenetic position of the species *S. kansuensis* (Fig. 6). The results show that *S. kansuensis* is a sister species to *S. lentigera* which, in turn, is also very similar in morphology, but differs in the larger thallus and by containing psoromic and 2’-O-demethypsoromic acids. *Squamarina nivalis* Frey & Poelt and *S. provincialis* Clauzade & Poelt are similar to *S. kansuensis* in having a strongly white pruinose thallus; however, *S. nivalis* differs in the smaller thallus, ca. 2 cm, not cracked upper surface, the apices of lobes bent downwards and the absence of psoromic acid; *S. provincialis* differs in the continuous but never overlapped lobes, the absence of the white thickened edges of lobes and the presence of atranorin. So far, the two species, *S. nivalis* and *S. provincialis*, are only known from very restricted places from Europe.

##### Specimens examined (all in KUN-L unless otherwise noted).

China: Gansu Province: Jiayuguan, 1500 m–1700 m elev., 1930, Briger Bohlin, S-L60805 (S); Yumen Ci., Moshan National Geological Park, 1760 m elev., 39°57'N, 97°14'E, on soil, 2018, Li-Song Wang et al. 18-59601; Sunan Co., Binggou Danxia landform Park, 1970 m elev., 38°56'N, 99°50'E, on soil, 2018, Li-Song Wang et al. 18-59658; Ningxia Province: Mt. Helanshan, 38°40'N, 1310 m elev., 105°46'E, on soil, 2014,Dong-Ling Niuet al. 14-09-1429 (NXAC); Qinghai Province: Wulan Co., Gobi desert along the way from Chaka to Wulan, 3151 m elev., 36°52'N, 98°55'E, on soil, 2018, Li-Song Wang et al. 18-59260, along the way from Wulan to Delingha, 3039 m elev., 36°59'N, 98°12'E, on soil, 2018, Li-Song Wang et al. 18-59274, 18-59306; Delingha Ci., Chayegou Station, 2974 m elev., 37°23'N, 96°37'E, on soil, 2018, Li-Song Wang et al. 18-59344, 18-59343. Sichuan Province: Derong Co., 1960 m elev., 28°12'N, 99°20'E, on soil, 2009, Li-Song Wang & Wang Jue 09-31112, 09-31118; Xizang Province: Linzhou Co., 3780 m elev., 29°54'N, 91°14'E, on soil, 2016, Li-Song Wang et al. 16-54052; Xinjiang Province: A-ke-tao Co., Oytagh observation zone, 2850 m elev., 38°54'N, 75°14'E, on soil, 2013, Hurnisa Shahidin et al. 20139103; Yunnan Province: Deqin Co., 2110 m elev., 28°13'N, 99°19'E, on soil, 2012, Li-Song Wang et al. 12-34756. Neimenggu Province: Beli-miao, 41°30'N, 110°10'E, on soil, 1929, Briger Bohlin, S-F304837 (S).

#### Squamarina oleosa

Taxon classificationFungiLecanoralesStereocaulaceae

(Zahlbr.) Poelt

1B3E068F-B340-58E5-923F-B416E050ADE3

Fig. 5H, I

Lecanora oleosa Zahlbr., in Handel-Mazzetti, *Symb. Sinic.* 3: 175 (1930) (Basionym) Type: China, Yunnan Province, Lijiang Co., Mt. Yulongxueshan, on rock, 1914, Heinrich Frh. von Handel-Mazetti 3576 (W–holotype!)

##### Description.

Thallus placodioid to subfoliose, rather closely attached to calcareous rocks, olive-green turning to yellowish-brown in the herbarium, up to 8 cm across and 5 mm high in the centre; lobes 2–4 mm long, 1.5–2.5 mm wide, ca. 1 mm thick, apices usually detached from the substrate with a white thickened edge; upper surface pruinose at least on the margins, matt to somewhat shiny, centrally cracked and faveolate-wrinkled, strongly convex, giving the thallus centre a bullate appearance, the base of the bullae carbonised, black; lower surface covered with pale brown to blackish-brown pulvinate hyphae, with sparse to numerous rhizinose strands; rhizinose strands brown to black, irregularly branched, up to 5 mm long. Upper cortex filled with yellowish-brown granules, turning colourless in KOH, 62–75 μm high, without epinecral; algal layer continuous, 65–70 μm thick; medulla filled with grey crystals of calcium oxalate and brick-red hyphae in lower part; lower cortex lacking.

Apothecia common but not abundant, laminal, scattered to slightly grouped, up to 4 mm in diam.; disc concave, plane to convex, light yellow, covered by yellowish pruina; thalline margin pruinose or not, darker than thallus, shiny, entire and distinctive when young, excluded with age. Hymenium 75–85 μm high, hyaline, I+ blue; epihymenium filled with yellowish-brown granules, not disperse into hymenium, turning colourless in KOH, 5–12.5 μm high; thalline margin without algae in the upper part, cortex filled with yellowish-brown granules, 112–125 μm thick; paraphyses septate, tips not swollen; hypothecium colourless, 100–162 μm thick, with pale brown granules forming a narrow line; algal layer below hypothecium continuous, 50–75 μm thick; ascus *Porpidia*-type, 8-spored. Ascospores ellipsoid to subfusiform, 15–20 × 5–7 μm. Pycnidia rare and small, ostioles yellow to yellowish-brown, conidia colourless, filiform, curved, 15–22.5 × ca. 0.7 μm.

##### Chemistry.

Upper cortex K-, C-, P-, medulla K-, C-, P+ yellow; usnic, psoromic and 2’-O-demethylpsoromic acids.

##### Ecology and distribution.

Growing on rock at elevations of 2623–3440 m. Only known from Yunnan Prov., China.

##### Notes.

The holotype grows on calcareous rock and bears only one apothecium.

This species was originally described as a *Lecanora* by Zahlbruckner (1930) and transferred to *Squamarina* by Poelt (1958). It is characterised by the thick, olive-green, placodioid to subfoliose thallus, yellowish apothecia covered with yellow pruina, the ellipsoid to subfusiform ascospores and the filiform, curved conidia. This species is the most basal clade in our reconstruction of the genus and it is close to *S. cartilaginea* and *S. gypsacea* (Fig. 6); however, *S. cartilaginea* differs in the non-pruinose, yellowish- to reddish-brown apothecia, smaller ascospores 10–14 × 4–6 μm and *S. gypsacea* differs in the yellowish-green, squamulose thallus, the very large and thick squamules that adnate to the substratum only by the central part and the larger apothecia (up to 1 cm). *Squamarina kansuensis* and *S. lentigera* can be distinguished from this species by the strongly white pruinose thallus, thinner lobes (< 0.5 mm) and smaller (< 2 mm) apothecia with non-pruinose and reddish-brown disc.

##### Specimens examined (all in KUN-L unless otherwise noted).

China: Yunnan Province: Lijiang Co., 3440 m elev., on rock, 2009, Li-Song Wang & Wang Jue 09-30034, Yulong Snow Mt., 26°56'N, 100°12'E, 2623 m elev., on calcareous rock, 2019, Li-Song Wang & Yan-yun Zhang 19-66398, 19-66399, 19-66401, 19-66402, 19-66404.

##### *Squamarina gypsacea* (O).

Greece: Corfu, hill above Troumpetas, 420 m elev., 39°74'N, 19°86'E, on exposed limestone outcrops, 2014, Rui, S. & Timdal, E., O-L-196249, Sokrati – Zigos road, 370 m elev., 39°72'N, 19°80'E, on rather shady limestone boulders in olive groove, 2014, Rui, S. & Timdal, E., O-L-196255; Kavalla, Thassos, along dirt road from Maries to Theologos, near Vatos, 590 m elev., 40°70'N, 24°66'E, on E-facing limestone wall in/above steep pine forest, 2000, Rui, S. & Timdal, E., O-L-59266. Spain: Alicante, between Callosa de Ensarria and Confrides, 260 m elev., 38°68'N, -0°21'E, 1985, Timdal, E., O-L-16444.

## Supplementary Material

XML Treatment for Lobothallia semisterilis

XML Treatment for Rhizoplaca callichroa

XML Treatment for Rhizoplaca pachyphylla

XML Treatment for Lecanora chondroderma

XML Treatment for Squamarina kansuensis

XML Treatment for Squamarina oleosa
